# Th1Th17_CM_ Lymphocyte Subpopulation as a Predictive Biomarker of Disease Activity in Multiple Sclerosis Patients under Dimethyl Fumarate or Fingolimod Treatment

**DOI:** 10.1155/2019/8147803

**Published:** 2019-06-26

**Authors:** Bibiana Quirant-Sánchez, Silvia Presas-Rodriguez, María José Mansilla, Aina Teniente-Serra, José V. Hervás-García, Luis Brieva, Ester Moral-Torres, Antonio Cano, Elvira Munteis, Juan Navarro-Barriuso, Eva M. Martínez-Cáceres, Cristina Ramo-Tello

**Affiliations:** ^1^Immunology Division, LCMN, Hospital Universitari Germans Trias i Pujol and Research Institute, Campus Can Ruti, Badalona, Barcelona, Spain; ^2^Department of Cellular Biology, Physiology and Immunology, Universitat Autònoma de Barcelona, Spain; ^3^Multiple Sclerosis Unit, Department of Neurosciences, Hospital Universitari Germans Trias i Pujol, Badalona, Barcelona, Spain; ^4^Neurology Department of Hospital Arnau Vilanova, Lleida, Spain; ^5^Neurology Department of Hospital San Joan Despi Moises Broggi, Barcelona, Spain; ^6^Neurology Department of Hospital de Mataró, Mataró, Barcelona, Spain; ^7^Neurology Department of Hospital del Mar, Barcelona, Spain

## Abstract

Peripheral blood biomarkers able to predict disease activity in multiple sclerosis (MS) patients have not been identified yet. Here, we analyzed the immune phenotype of T lymphocyte subpopulations in peripheral blood samples from 66 RRMS patients under DMF (*n* = 22) or fingolimod (*n* = 44) treatment, by flow cytometry. A correlation study between the percentage and absolute cell number of each lymphocyte subpopulation with the presence of relapses or new MRI lesions during 12-month follow-up was performed. Patients who had undergone relapses showed at baseline higher percentage of Th1_CM_ cells (relapsed: 11.60 ± 4.17%*vs*. nonrelapsed: 9.25 ± 3.17%, *p* < 0.05) and Th1Th17_CM_ cells (relapsed: 15.65 ± 6.15%*vs*. nonrelapsed: 10.14 ± 4.05%, *p* < 0.01) before initiating DMF or fingolimod treatment. Kaplan-Meier analysis revealed that patients with Th1Th17_CM_ (CD4^+^CCR7^+^CD45RA^−^CCR6^+^CXCR3^+^) cells > 11.48% had a 50% relapse-free survival compared to patients with Th1Th17_CM_cells < 11.48% whose relapse-free survival was 88% (*p* = 0.013, log-rank test). Additionally, a high percentage of Th1Th17_CM_ cells was also found in patients with MRI activity (MRI activity: 14.02 ± 5.87%*vs*. no MRI activity: 9.82 ± 4.06%, *p* < 0.01). Our results suggest that the percentage of Th1Th17_CM_ lymphocytes at baseline is a predictive biomarker of activity during the first 12 months of treatment, regardless of the treatment.

## 1. Introduction

Multiple sclerosis (MS) is a chronic autoimmune inflammatory disease of the central nervous system (CNS), characterized by immune cell infiltration, demyelination, axonal degeneration, and astrogliosis [1, 2].

The CD4^+^ T-helper (Th) lymphocytes are the most studied cell subpopulation in MS. Different authors have demonstrated that T-helper 1 (Th1), T-helper 17 (Th17), and T-helper 1/17 (Th1Th17; also referred as Th1-like Th17 in the literature) CD4^+^ T cells promote or contribute to the autoimmune inflammatory process of MS patients and its mouse model, the experimental autoimmune encephalomyelitis (EAE) [3–7]. Th1Th17 cells are characterized by the production of proinflammatory cytokines such as IFN-*γ* (Th1) and IL-17 (Th17). Additionally, *in vitro* studies showed that human Th1Th17 lymphocytes with memory phenotype (CD4^+^CD45RO^+^IL-17A^+^) migrate more avidly across the blood-brain barrier than Th1 cells [4]. These results were also confirmed *in vivo*, by immunohistofluorescence studies of the CNS lesions of mice with EAE, indicating that Th1Th17 cells are more pathogenic than Th1 lymphocytes, although both Th1 and Th17 are required for EAE induction [5].

Effective prevention of MS relapses partially reduces accumulation of neurological disability [8]. Nowadays, disease-modifying therapies (DMTs) are the standard treatment for patients with RRMS and these treatments are postulated to reduce clinical relapses and radiological activity, through the reduction of the activation, migration, or differentiation of different lymphocyte subpopulations [8, 9]. Despite these beneficial effects, clinical and radiological outcomes remain highly variable among patients. Thereby, there is an unmet need to identify objective and accessible biomarkers of activity in MS patients [10, 11].

Dimethyl fumarate (DMF) is an oral treatment for RRMS, able to reduce the relapse rate and the number of new or enlarging MRI lesions [12]. Although its mechanisms of action is not yet well understood, it is postulated that DMF induces a switch toward a Th2 cytokine profile and a reduction in the migratory capacity of the immune cells by the inhibition of nuclear factor kappa B (NF-*κ*B) [13]. In addition, it induces activation of nuclear factor erythroid 2-related factor 2 (Nrf-2), with neuroprotective and antioxidant properties [14].

Another oral treatment for RRMS patients is fingolimod, a sphingosine 1-phosphate receptor modulator that reduces the egress of lymphocytes from lymph nodes and, consequently, the infiltration of potentially autoreactive lymphocytes into the CNS [15].

The effect of both treatments on lymphocyte subpopulations has been described, showing relevant changes of these cells in peripheral blood of patients treated with these drugs [16–21]. While fingolimod mainly reduces CCR7^+^ cells—naïve and central memory T cells—which are regulated by homing signals to lymph nodes [22], DMF induces a reduction of effector T cells, as well as a decrease of CD4^+^ T cells that express interferon-*γ* (IFN-*γ*) and interleukin-17 (IL-17) [23, 24].

There are no studies relating the basal levels of lymphocyte subpopulations with the risk to develop relapses or MRI lesions during treatment. In our study, we analyzed peripheral blood T cell subpopulations in RRMS patients at baseline by flow cytometry and correlated their levels to outcome parameters (relapses and MRI activity) appearing during the first 12 months of DMF or fingolimod treatment.

## 2. Methods

### 2.1. Patients

Sixty-six RRMS patients fulfilling the 2010 McDonald's criteria [25] who started DMF (*n* = 22) or fingolimod (*n* = 44) treatment were included. Patients were classified into active or nonactive according to relapses and MRI activity (Table 1) over a follow-up period of 12 months. Patients who had progressive forms of MS or RRMS patients receiving interferon beta (IFN-*β*) or glatiramer acetate (GA) within the previous 24 h, or fingolimod in the previous 15 days, or natalizumab (NTZ), teriflunomide, diazoxide, or methylprednisolone within the previous 30 days prior to the start of the DMF or fingolimod treatment were excluded from the study.

Relapses were defined as new or worsening neurological deficit lasting 24 h or more in the absence of fever or infection. The annualized relapse rate (ARR) was defined as the total number of relapses divided by the number of patients-year assessed in the 12 months prior the initiation of treatment (baseline) and after 12-month follow-up.

MRI activity was measured by any new or enlarging T2 lesions and/or gadolinium enhancement T1 lesions (Gd+) in brain MRI. A 1.5 or 3.0 Tesla MRI scans were used to evaluate the number of lesions before and after 12 months of treatment. The same equipment was used in each patient. The number of lesions was determined visually comparing the first and second MRI scans.

Disability progression was defined as worsening of 1 point or more on the expanded disability status scale (EDSS) score over the baseline.

### 2.2. Flow Cytometry Analysis

Blood samples were collected prior to the first administration of the treatment (baseline) and processed by a centralized laboratory within the first 24 h after their collection. Samples of whole blood were analyzed by flow cytometry to determine the percentage and absolute number of T lymphocyte subpopulations using the following combination of monoclonal antibodies per panel: CD3-V450, CD4 PerCP-Cy5.5, CD45RA PE-Cy7, CCR7 PE, CD38 APC, CD8 APC-H7, HLA-DR V500 (BD Biosciences), CD183 AF488, CD196 BV605, and CD45 AF700 (BioLegend, San Diego, CA, USA). The absolute cell number quantification was performed as previously reported [16]. Samples were acquired on a LSR II Fortessa flow cytometer (BD Biosciences, San José, CA, USA).

**Figure 1:**
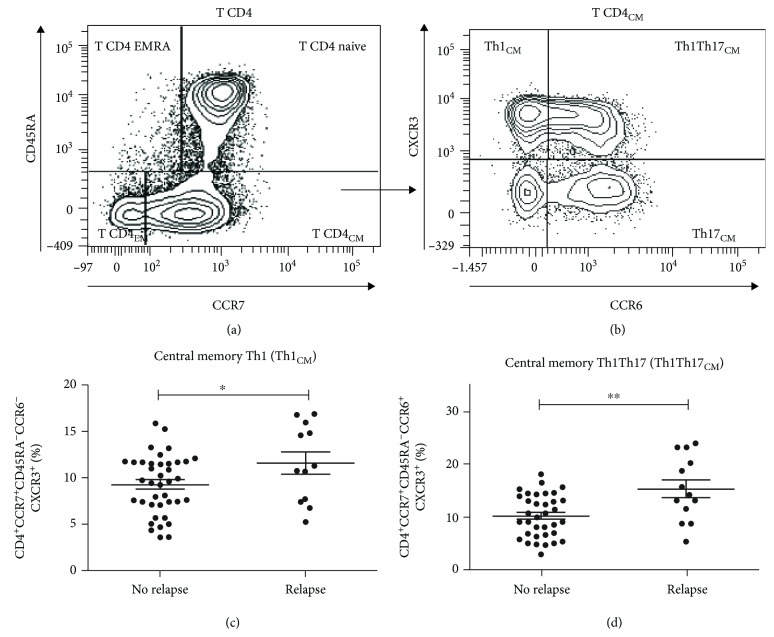
Distribution of lymphocyte subpopulations at baseline in MS patients who presented clinical relapses during the first year of treatment. Representative example of the flow cytometry gating strategy used to analyze the different stages of maturation CD4^+^ T lymphocyte subpopulations (a) and central memory Th1, Th2, Th17, and Th1Th17 lymphocytes (b). Percentage of Th1 central memory cells (Th1_CM_) (*n* = 53) (c) and percentage of Th1Th17 central memory cells (Th1Th17_CM_) (*n* = 53) (d). Each dot represents the value of an individual patient. CM = central memory; EM = effector memory. ^∗^ *p* < 0.05; ^∗∗^ *p* < 0.01.

The following T cell subpopulations were analyzed: CD4^+^ naïve, CD4^+^ T_CM_, Th1_CM_, Th1Th17_CM_, Th2_CM_, Th17_CM_, CD4^+^ T_EMRA_, CD4^+^ T_EM_, Th1_EM_, Th1Th17_EM_, Th2_EM_, Th17_EM_, CD8^+^ naïve, CD8^+^ T_CM_, CD8^+^ T_EMRA_, CD8^+^ T_EM_, double positive (CD4^+^CD8^+^), and double negative (CD4^−^CD8^−^) T cells. Analysis was performed using the FACSDiva software (BD Biosciences). The gating strategy for the subpopulations analyzed in whole blood is shown in Figure 1 and previously described by Quirant-Sánchez et al. [22].

### 2.3. Statistical Analysis

The comparisons of the clinical characteristics of patients were carried out using the Wilcoxon test to compare two groups in the case of paired data. The Kruskal-Wallis test was used to compare more than three groups in the case of independent data. For the analysis of the relapse and MRI activity groups, mean baseline values of each group were compared using the nonparametric unpaired *t*-test (Mann-Whitney test). Predictive values of the percentages and absolute number of lymphocyte subpopulations were investigated through receiver operating characteristic (ROC) curves. Based on these curves, cut-off values for relapse prediction were assessed for each potential biomarker. For these biomarkers, Kaplan-Meier curves with the log-rank test were used to test differences of survival probability between groups of patients. Values of *p* < 0.05 were considered statistically significant. Results are expressed as means ± SEM. The Statistical Package for Social Sciences (SPSS/Windows version 15.0; SPSS Inc., Chicago, IL, USA) and the software program GraphPad Prism (5.0 version; GraphPad, La Jolla, CA, USA) were used to perform statistical analyses.

## 3. Results

### 3.1. Patients

Clinical and demographical characteristics of patients are shown in Table 1. No differences were found at baseline between groups.

From the sixty-six RRMS, 44 patients were treated with fingolimod and 22 patients with DMF. After 12 months, 60% of patients treated with fingolimod and 83% of patients treated with DMF did not experience relapses (nonrelapsed patients). A total of 21 patients underwent at least one relapse during follow-up, and 57% of them (12 of 21 patients) presented a relapse in the first three months after starting treatment.

Although most of the patients did not receive any DMT previously (33 naïve patients), patients treated with previous immunomodulatory treatments were included in our study. The distribution of a previous treatment in the cohort of fingolimod was 18 naïve patients, 12 switched from IFN-*β*, 10 from NTZ (all for positive JC virus serostatus), 3 from GA, and only one patient switched from diazoxide. In the cohort of DMF, the following patients were included: 15 naïve patients, 4 switched from IFN-*β*, 1 patient switched from teriflunomide, and 1 from fingolimod. In addition, one patient who had participated in a clinical trial with mesenchymal stem cells was included. No other previous treatments were included in this study (Table 1).

According to a previous treatment, the distribution of patients who did not undergo relapses during the follow-up was (i) 64% of 33 naïve patients, (ii) 69% of 16 IFN-*β* patients, (iii) 70% of 10 NTZ patients, and (iv) 68% of 3 GA patients (Table 1).

The analysis of the ARR according to the treatment started during the follow-up is shown in Table 2. Previous DMTs and washout period were considered in the ARR analysis. No differences in the distribution of patients depending on the previous treatment were found in relation to the ARR at baseline (Table 2). The ARR was significantly reduced during the 12-month follow-up period (0.5 ± 0.8) vs. (1.53 ± 1.1) (*p* < 0.001). Particularly, patients who had received a previous treatment had shown a reduction in the ARR from 1.6 ± 0.93 to 0.42 ± 0.75 (*p* < 0.001) and naïve patients showed a reduced ARR from 1.42 ± 1.32 to 0.55 ± 0.77 (*p* < 0.01).

### 3.2. Increase of Th1_CM_ and Th1Th17_CM_ Cells in Patients That Presented Clinical Relapses

We compared the percentage and absolute number of T lymphocyte subpopulations (Table 3), at baseline, of patients that experienced at least one clinical relapse (*n* = 21) with those that remained relapse-free (*n* = 45). Although the percentage of CD4^+^ T_CM_ cells did not show differences between groups in the total cohort of patients (Table 4), a deep analysis of minor subpopulations of CD4^+^ T_CM_ pointed out a significantly higher percentage Th1_CM_ cells (CD4^+^CCR7^+^CD45RA^−^CCR6^−^CXCR3^+^) and Th1Th17_CM_ cells (CD4^+^CCR7^+^CD45RA^−^CCR6^+^CXCR3^+^) at baseline in patients that underwent relapses (Th1: relapsed: 11.60 ± 4.17%*vs*. nonrelapsed: 9.25 ± 3.17%, *p* < 0.05, Figure 1(c); Th1Th17: relapsed: 15.65 ± 6.15%*vs*. nonrelapsed: 10.14 ± 4.05%, *p* < 0.01, Figure 1(d) and Figure S1). Th1_CM_ and Th1Th17_CM_ cells from 13 patients were not available from the study. No statistically differences were observed in Th1_CM_ and Th1Th17_CM_ lymphocytes between patients treated with DMF or fingolimod at baseline.

Only one DMF patient and three fingolimod patients experienced relapses in the last 6 months of follow-up. These patients did not show differences in the percentage of Th1Th17_CM_ at baseline (relapsed in the last 6 months of follow-up: 14.15 ± 2.02; *p* = 0.2482*vs*. relapsed in the first 6 months of follow-up: 18.33 ± 1.78) and Th1_CM_ (relapsed in the last 6 months of follow-up 12.16 ± 1.42*vs*. relapsed in the first 6 months of follow-up 11.01 ± 2.9; *p* = 0.70) compared to patients who experienced relapses in the first 6 months starting the treatment.

The analysis of the percentage of CD4^+^ T_CM_ in the fingolimod cohort showed a higher percentage in patients who experienced relapses during the 12 months of treatment (relapsed: 41.28 ± 13.84%*vs*. nonrelapsed: 36.38 ± 9.83%, *p* = 0.034) (Table S1). No differences were found in the percentage of CD4^+^ T_CM_ in the DMF cohort.

Patients in which the prior treatment was NTZ showed a higher percentage of CD4^+^ T_CM_ at baseline than naïve patients (NTZ-treated patients: 47.4 ± 5.6%*vs*. naïve patients: 37.56 ± 11.09%, *p* = 0.013). A deep analysis of CD4^+^ T_CM_ cell subsets—Th1_CM_, Th17_CM_, and Th1Th17_CM_—revealed no differences at baseline between naïve and patients previously treated with DMT as well as no differences between naïve and patients that switched from NTZ (Th1_CM_: NTZ patients: 10.39 ± 3.36%*vs*. naïve patients: 9.83 ± 3.35%, *p* = 0.64, Th17_CM_: NTZ patients: 13.65 ± 2.9%*vs*. naïve patients: 10.46 ± 5.45%, *p* = 0.09, and Th1Th17_CM_: NTZ patients: 14.56 ± 5.36%*vs*. naïve patients: 12.65 ± 6.42%, *p* = 0.39).

In relation to absolute numbers of lymphocyte subpopulations, no differences of Th1_CM_, Th17_CM_, and Th1Th17_CM_ were found. In contrast, the absolute number of CD4^+^ T_CM_ cells at baseline was higher in patients who experienced relapses during the follow-up (*p* < 0.05) (Table 4).

**Figure 2:**
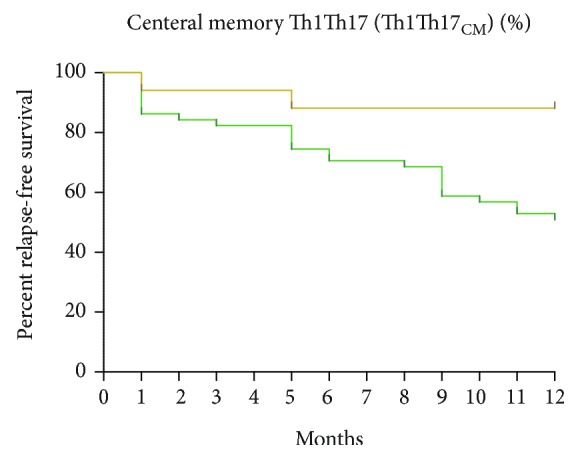
Relapse-free survival for patients within the first 12 months after treatment. Groups were separated by percentage of Th1Th17_CM_ out of CD4^+^ T cells. Patients with less than 11.48% of Th1Th17_CM_ (*n* = 26) and more than 11.48% (*n* = 27) (*p* < 0.01, log-rank test).

To determine whether elevated percentages of Th1Th17_CM_ cells at baseline were associated with an increased risk to develop relapses, we analyzed the outcome of patients after 12-month follow-up. We defined ROC curves that enabled us to identify values of Th1Th17_CM_ percentages in our cohort of patients, predicting the risk to undergo relapses. Baseline values of Th1Th17_CM_ > 11.48% indicated an increased risk to develop relapse (sensibility of 75%, specificity of 62%, area under curve (AUC) of 0.74, *p* < 0.04). Then, the ability to predict relapse-free survival of the potential biomarker was compared. Kaplan-Meier curves showed that patients with Th1Th17_CM_ > 11.48% (*n* = 27) showed a relapse-free survival of 50% compared to 88% for patients with Th1Th17_CM_ < 11.48% (*n* = 26) (*p* < 0.01, log-rank test) (Figure 2 and Figure S2).

### 3.3. Th1Th17_CM_ Lymphocytes as a Prognostic Factor for MRI Activity

A total of 33% of patients had MRI activity at 12 months of follow-up under DMF or fingolimod treatment. Almost all patients (*n* = 58) had a MRI available. The distribution of patients who presented MRI activity based on previous treatments was 33% of naïve patients, 38% of IFN-*β* patients, and 22% of NTZ. Fourteen patients under fingolimod treatment and four patients treated with DMF showed MRI activity after 12-month follow-up.

Given that we previously observed a high percentage of Th1Th17_CM_ cells in clinically active MS patients, we studied the association of MRI activity with this lymphocyte subpopulation.

A higher percentage of Th1Th17_CM_ cells was found in patients with MRI activity (MRI activity: 14.02 ± 5.87%, *p* < 0.01; no MRI activity: 9.82 ± 4.06%). No differences were found either in the percentage of Th1_CM_, Th17_CM_, and CD4^+^ T_CM_ lymphocytes or in the absolute number of the lymphocyte subpopulations studied, including Th1Th17_CM_ lymphocytes (Table 4).

## 4. Discussion

Patients with RRMS have a large number of treatment options available. There is a need to investigate objective and accessible biomarkers able to predict treatment response. Predictive biomarkers of disease activity would help to choose initial therapy, monitoring response to therapy and detecting subclinical disease activity. The present study proposes that, regardless of the treatment, the increased percentage of Th1Th17_CM_ lymphocytes at baseline could be a predictive biomarker of relapses or MRI activity during the first 12 months of treatment.

The relapse process in nontreated MS patients is linked to the presence of CD4^+^ T_CM_ and Th1Th17_CM_ lymphocytes in peripheral blood and CNS [5, 6, 21]. The Th1Th17 lymphocytes are characterized by an activated phenotype and higher migratory capacity to CNS. Actually, a recent study described Th1Th17_CM_ lymphocytes as key regulators in the onset of MS due to their predominance in the CD4^+^ T cell pool of peripheral blood in early stages of disease [7]. Additionally, Sato et al. described an increase in Th1Th17 cells at the time of the MS relapses in patients following fingolimod treatment [21]. Accordingly, our findings showed a significant higher percentage of Th1_CM_ and Th1Th17_CM_ in peripheral blood at baseline in patients who experienced relapses during the 12-month follow-up. In addition, we found a statistically significant association on the percentage of Th1Th17_CM_ lymphocytes with MRI activity. These results suggest an important role of these cells in MS pathogenesis and support the idea that they could be a potential predictive biomarker of disease activity.

Most of the mechanisms of action of DMTs are addressed to reduce autoreactive and proinflammatory immune mechanisms in MS and thus are used to reduce the number of relapses [12–15]. Although the immunological effect of these treatments reducing the number of lymphocytes in peripheral blood is already detectable after the first month of treatment, in some patients, its efficacy is observed only after the third or sixth month of treatment [16–21, 26]. In this context, the analysis of the percentage of Th1Th17_CM_ and Th1_CM_ lymphocytes at baseline from patients who experienced relapses in the last 6 months of follow-up did not show differences compared to patients who experienced relapses in the first 6 months starting treatment. More information at different time points is necessary to determine the relevance of these biomarkers at the time of the relapse during the treatment.

An important issue to take into account is that the currently established washout periods for patients with previous treatments could be insufficient to achieve a proper recovery of the normal immune profile. In fact, in contrast to other DMTs, patients that switched from NTZ showed higher CD4^+^ T_CM_ percentages at baseline than naïve patients. However, those patients remained with the same ARR after fingolimod treatment, which has also been observed by other authors [27]. For this reason, the percentage of CD4^+^ T_CM_ cells would not constitute a useful biomarker in cases of patients previously treated with NTZ. Interestingly, the percentage of Th1Th17_CM_ could be used as a predictive biomarker in those patients, as the distribution of CD4^+^ Th1_CM_, Th17_CM_, and Th1Th17_CM_ subsets at baseline was not affected by the effect of a previous NTZ treatment.

Although our results showed a trend of higher percentages of Th1Th17_CM_ lymphocytes at baseline in DMF patients who develop at least one relapse during the first 12 months of follow-up, our study presents an important limitation in the number of DMF patients who had relapses. A large confirmatory cohort will be necessary to validate these results. Nonetheless, our study suggests that the analysis monitoring of the Th1Th17_CM_ subpopulation at baseline may be useful in order to personalize therapeutic approaches and prevent relapses in MS patients.

## 5. Conclusion

We have identified Th1Th17_CM_ cells as a lymphocyte subpopulation increased at baseline in peripheral blood of patients with a higher risk to develop relapses or new MRI lesions during the first year of treatment with DMF or fingolimod.

## Figures and Tables

**Table 1:** Clinical and demographic characteristics of MS patients included in the study.

	Total cohort (*n* = 66)	Relapsed (*R*) (*n* = 21)	Nonrelapsed (NR) (*n* = 45)	DMF cohort (*n* = 22)	FNG cohort (*n* = 44)
Female sex (no. of patients (%))	42 (63)	12 (57)	30 (67)	15 (68)	26 (59)

Age (years), mean (SD)	35.29 ± 8.5	32.62 ± 7.66	36.5 ± 8.67	36.7 ± 7.4	34.9 ± 8.9

First symptoms (years), mean (SD)	4.72 ± 5.47	4.51 ± 6.08	4.81 ± 5.25	4.18 ± 4.72	4.7 ± 5.9

Previous immunomodulatory drugs	33 naïve	11 naïve	22 naïve	15 naïve	18 naïve
	16 IFN-*β*	4 IFN-*β*	12 IFN-*β*	4 IFN-*β*	12 IFN-*β*
	3 GA^∗^	1 GA^∗^	2 GA^∗^		3 GA^∗^
	10 NTZ^∗^	3 NTZ^∗^	7 NTZ^∗^		10 NTZ^∗^
	1 diazoxide		1 diazoxide		1 diazoxide
	1 teriflunomide			1 teriflunomide	
	1 fingolimod			1 fingolimod	
	1 others^∗^			1 others^∗^	

Number of previous treatments (years), mean (SD)	1 ± 1	1 ± 1.3	0.5 ± 0.8	0.83 ± 0.96	0.83 ± 0.98
0 treatment	33	13	20	15	18
1 treatment	16	4	12	4	12
2 treatments	12	5	7	1	11
≥3 treatments	5	2	3	2	3

ARR^∗^ previous year					
Total patients	1.53 (1.1)	1.53 (1.2)	1.52 (0.8)	1.14 (0.7)	1.53 (1.13)
Naïve patients	1.54 (0.9)	1.55 (1.1)	1.6 (0.8)	1.6 (0.8)	1.61 (1.20)
Treated patients	1.5 (1.3)	1.6 (1.5)	1.3 (1)	1.3 (1)	1.52 (1.21)

^∗^DMF: dimethyl fumarate; FNG: fingolimod; IFN-*β*: interferon beta; GA: glatiramer acetate; NTZ: natalizumab; others: clinical trial of cell therapies; ARR: annualized relapse rate; SD: standard deviation.

**Table 2:** Analysis of clinical and radiologic characteristics of the patients after 12 months of treatment.

	DMF cohort (*n* = 22)			Fingolimod cohort (*n* = 44)		
	Baseline	+12 months	*p* value	Baseline	+12 months	*p* value
ARR^∗^ mean (SD)						
Total patients	1.5 (1.01)	0.14 (0.35)	*p* < 0.0001	1.56 (1.18)	0.61 (0.84)	*p* < 0.0001
Naive patients	1.6 (0.8)	0.06 (0.26)	*p* < 0.0001	1.72 (1.01)	0.83 (0.86)	*p* < 0.01
Treated patients	1.6 (1.4)	0.28 (0.48)	*p* < 0.05	1.4 (1.35)	0.46 (0.81)	*p* < 0.01
Relapse-free patients (no. of patients (%))		19 (86.4)			25 (62.5)	
Progression-free patients (no. of patients (%))		21 (95)			34 (85)	
Free MRI activity (no. of patients (%))		15 (79)			25 (64)	

^∗^DMF: dimethyl fumarate; ARR: annualized relapse rate.

**Table 3:** Phenotype of T cell subpopulations by flow cytometry.

Lymphocyte subpopulations	Phenotype
*T cell subsets (CD3^+^)*	
CD4^+^ naïve T cell	CD4^+^CCR7^+^CD45RA^+^
CD8^+^ naïve T cell	CD8^+^CCR7^+^CD45RA^+^
*CD4^+^ central memory (CD4^+^ T_CM_)*	CD4^+^CCR7^+^CD45RA^−^
Th1 central memory (Th1_CM_)	CD4^+^CCR7^+^CD45RA^−^CCR6^−^CXCR3^+^
Th2 central memory (Th2_CM_)	CD4^+^CCR7^+^CD45RA^−^CCR6^−^CXCR3^−^
Th17 central memory (Th17_CM_)	CD4^+^CCR7^+^CD45RA^−^CCR6^+^CXCR3^−^
Th1Th17 central memory (Th1Th17_CM_)	CD4^+^CCR7^+^CD45RA^−^CCR6^+^CXCR3^+^
CD8+ central memory T cell (CD8^+^ T_CM_)	CD8^+^CCR7^+^CD45RA^−^
*CD4^+^ effector memory T cell (CD4^+^ T_EM_)*	CD4^+^CCR7^−^CD45RA^−^
Th1 effector memory (Th1_EM_)	CD4^+^CCR7^−^CD45RA^−^CCR6^−^CXCR3^+^
Th2 effector memory (Th2_EM_)	CD4^+^CCR7^−^CD45RA^−^CCR6^−^CXCR3^−^
Th17 effector memory (Th17_EM_)	CD4^+^CCR7^−^CD45RA^−^CCR6^+^CXCR3^−^
Th1Th17 effector memory (Th1Th17_EM_)	CD4^+^CCR7^−^CD45RA^−^CCR6^+^CXCR3^+^
CD8^+^ effector memory T cell (CD8^+^ T_EM_)	CD8^+^CCR7^−^CD45RA^−^
Terminal differentiated effector memory CD4^+^ T cell (T_EMRA_)	CD4^+^CCR7^−^CD45RA^+^
Terminal differentiated effector memory CD8^+^ T cell (T_EMRA_)	CD8^+^CCR7^−^CD45RA^+^
Double negative T cell	CD4^−^CD8^−^
Double positive T cell	CD4^+^CD8^+^

**Table 4:** Differences in the percentage and absolute number of central memory T cell subpopulations at baseline according to the clinical outcome in dimethyl fumarate- or fingolimod-treated patients.

Lymphocyte subpopulations at baseline	Total cohort (*n* = 66)		DMF (*n* = 22)		Fingolimod (*n* = 44)	
	MRI activity (no MRI activity, *n* = 40)	Relapses (nonrelapsed, *n* = 45)	MRI activity (no MRI activity, *n* = 15)	Relapses (nonrelapsed, *n* = 19)	MRI activity (no MRI activity, *n* = 25)	Relapses (nonrelapsed, *n* = 25)
	*p* value	*p* value	*p* value	*p* value	*p* value	*p* value
CD4^+^ T_CM_ (%)	0.147	0.154	0.057	0.561	0.609	**0.034**
Th1_CM_ (%)	0.326	**0.049**	0.505	0.289	0.637	0.093
Th17_CM_ (%)	0.878	0.06	0.502	0.557	0.477	0.345
Th1Th17_CM_ (%)	**0.006**	**0.002**	0.09	0.842	0.062	**0.040**
CD4^+^ T_CM_ (cel/*μ*L)	0.515	**0.048**	0.057	0.980	0.998	**0.020**
Th1_CM_ (cel/*μ*L)	0.150	0.877	0.088	0.443	0.679	0.059
Th17_CM_ (cel/*μ*L)	0.156	0.746	0.440	0.391	0.235	0.134
Th1Th17_CM_ (cel/*μ*L)	0.168	0.190	0.192	0.540	0.512	0.052

DMF: dimethyl fumarate; CD4^+^ T_CM_: central memory CD4 T lymphocytes; Th1_CM_: Th1 central memory lymphocytes; Th17_CM_: Th17 central memory lymphocytes; Th1Th17_CM_: Th1Th17 central memory lymphocytes; MRI: magnetic resonance imaging. *p* values in bold indicate statistically significance.

## Data Availability

The flow cytometry data used to support the findings of this study are included within the article, including the values behind the means, standard deviations, and other measures reported.

## Supplementary Materials

Supplementary Materials Table S1: percentage of central memory T cell subpopulations in relation to clinical outcome parameters (MRI activity and relapses) in RRMS patients. Figure S1: distribution of Th1CM and Th1Th17_CM_ lymphocyte subpopulations at baseline in MS patients from DMF (a, b) and fingolimod cohort (c, d), respectively. Figure S2: relapse-free survival curve for patients during the first 12 months of DMF treatment (a), fingolimod treatment (b), and total cohort of patients (c). Groups were separated by percentage of Th1Th17_CM_ out of CD4^+^ T cells. Patients with less than 11.48% of Th1Th17_CM_ (*n* = 26) and more than 11.48% (*n* = 27) ((a) *p* = 0.27; (b) *p* = 0.08; (c) *p* < 0.01, log-rank test).
