# Supplementary material for: Th1Th17CM Lymphocyte Subpopulation as a Predictive Biomarker of Disease Activity in Multiple Sclerosis Patients under Dimethyl Fumarate or Fingolimod Treatment
Source: Mediators Inflamm. 2019 Jun 26;2019:8147803. doi: 10.1155/2019/8147803 (PMC6617925; doi:10.1155/2019/8147803)
Supplement: Supplementary Materials — Table S1: percentage of central memory T cell subpopulations in relation to clinical outcome parameters (MRI activity and relapses) in RRMS patients. Figure S1: distribution of Th1CM and Th1Th17CM lymphocyte subpopulations at baseline in MS patients from DMF (a, b) and fingolimod cohort (c, d), respectively. Figure S2: relapse-free survival curve for patients during the first 12 months of DMF treatment (a), fingolimod treatment (b), and total cohort of patients (c). Groups were separated by percentage of Th1Th17CM out of CD4+ T cells. Patients with less than 11.48% of Th1Th17CM (n = 26) and more than 11.48% (n = 27) ((a) p = 0.27; (b) p = 0.08; (c) p < 0.01, log-rank test). [file 8147803.f1.docx]

**Table S1. Percentage of central memory T cell subpopulations in relation to clinical outcome parameters (MRI activity and relapses) in RRMS patients.**

| Lymphocyte subpopulations | MRI activity | Mean % | Std error | *p* value | Relapses | Mean | Std error | *p* value |
| --- | --- | --- | --- | --- | --- | --- | --- | --- |
| CD4^+^ T_CM_ % | No activity | 36.17 | 10.43 | 0.147 | No relapse | 36.29 | 10.04 | 0.154 |
|  | Activity | 40.55 | 12.71 |  | Relapse | 40.72 | 13.69 |  |
| Th1 _CM_ % | No activity | 10.10 | 3.68 | 0.326 | No relapse | 9.25 | 3.17 | **0.049** |
|  | Activity | 9.005 | 3.81 |  | Relapse | 11.60 | 4.17 |  |
| Th17 _CM_ % | No activity | 10.18 | 4.16 | 0.878 | No relapse | 9.71 | 3.44 | 0.06 |
|  | Activity | 10.35 | 3.56 |  | Relapse | 11.96 | 4.87 |  |
| Th1Th17 _CM_ % | No activity | 9.82 | 4.05 | **0.006** | No relapse | 10.14 | 4.05 | **0.002** |
|  | Activity | 14.02 | 5.87 |  | Relapse | 15.65 | 5.87 |  |
| CD4^+^ T_CM_ cel/µL | No activity | 358.9 | 225.8 | 0.515 | No relapse | 325.6 | 167.3 | **0.048** |
|  | Activity | 327.6 | 193.2 |  | Relapse | 379.8 | 257.3 |  |
| Th1 _CM_ cel/µL | No activity | 52.04 | 45.95 | 0.150 | No relapse | 59.44 | 53.45 | 0.877 |
|  | Activity | 64.97 | 53.83 |  | Relapse | 53.08 | 39.28 |  |
| Th17 _CM_ cel/µL | No activity | 57.57 | 36.11 | 0.156 | No relapse | 59.38 | 51.84 | 0.746 |
|  | Activity | 77.14 | 61.54 |  | Relapse | 54.79 | 35.04 |  |
| Th1Th17 _CM_ cel/µL | No activity | 54.80 | 10.77 | 0.168 | No relapse | 51.24 | 51.61 | 0.190 |
|  | Activity | 78.49 | 60.61 |  | Relapse | 75.32 | 59.70 |  |

CD4^+^ T_CM_: Central memory CD4 T lymphocytes; Th1 _CM_: Th1 Central memory lymphocytes; Th17 _CM_: Th17 Central memory lymphocytes; Th1Th17 _CM_: Th1Th17 Central memory lymphocytes; MRI: magnetic resonance imaging. *P*-values in bold indicate statistically significance.


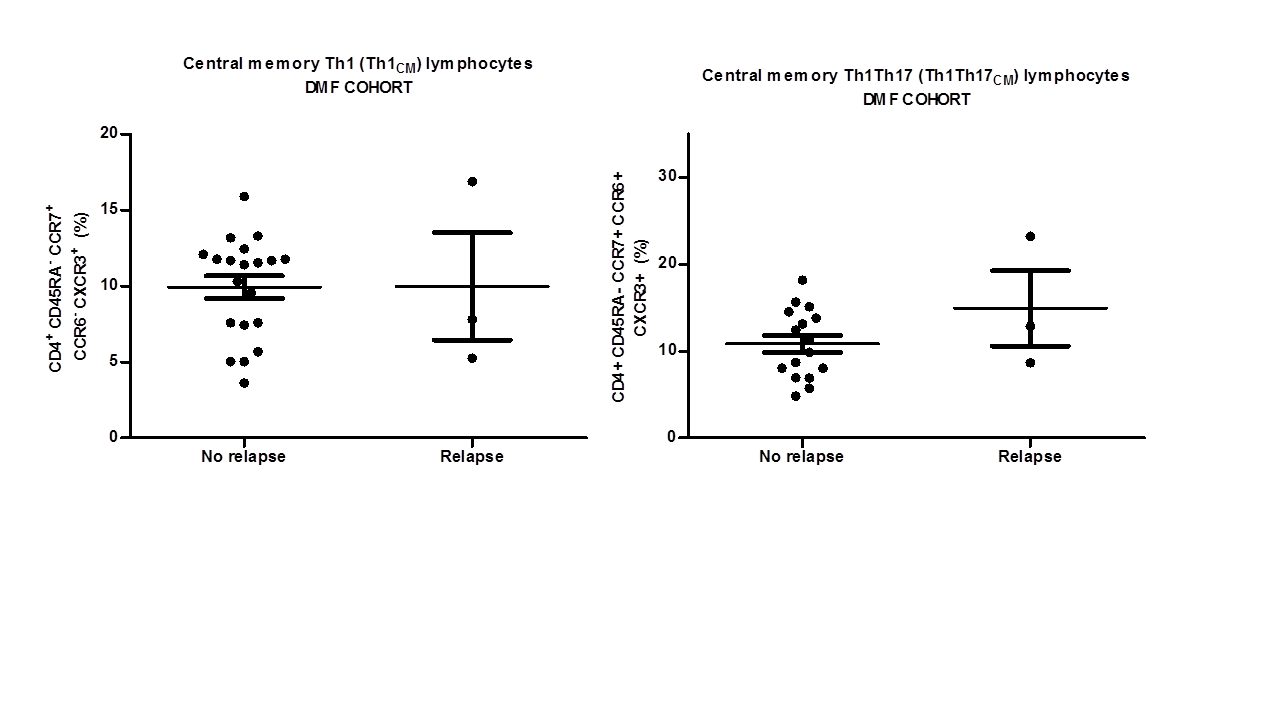


b)

a)


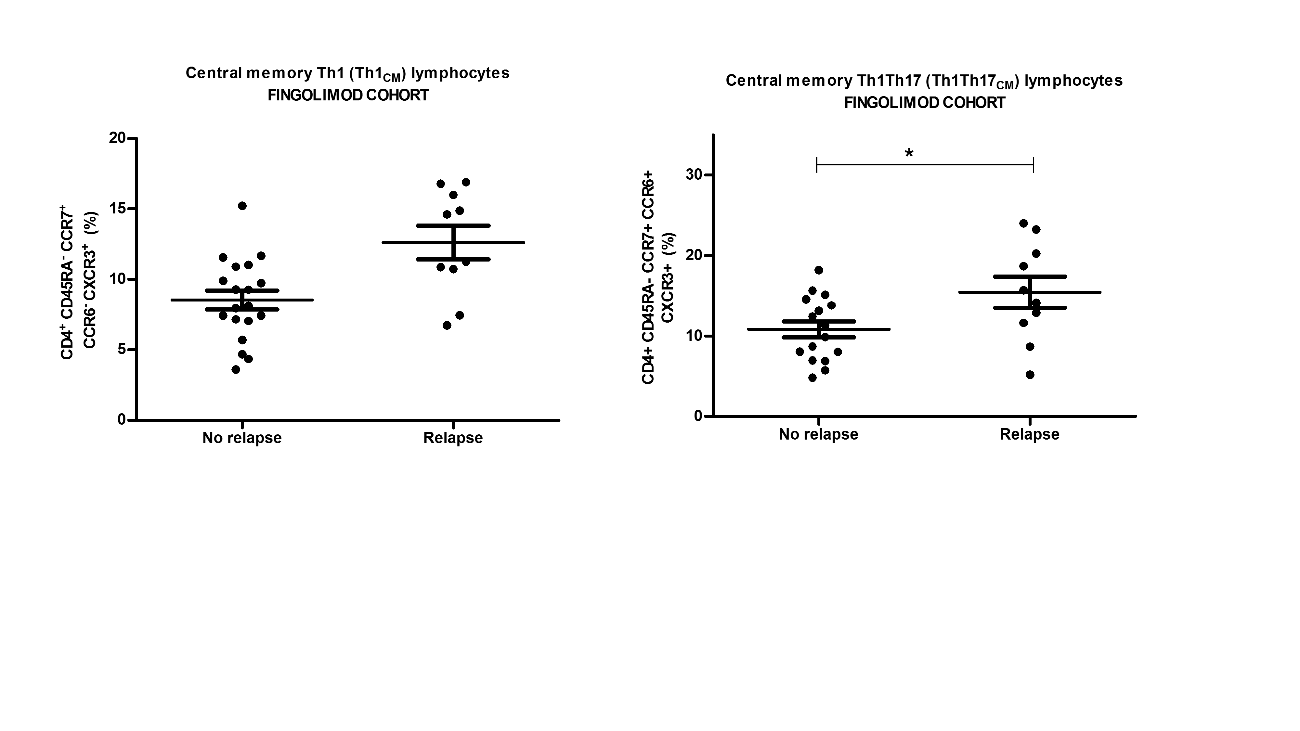


d)

c)

**Figure S1.** Distribution of Th1_CM_ and Th1Th17_CM_ lymphocyte subpopulations at baseline in MS patients from DMF (a,b) and fingolimod cohort (c,d), respectively.


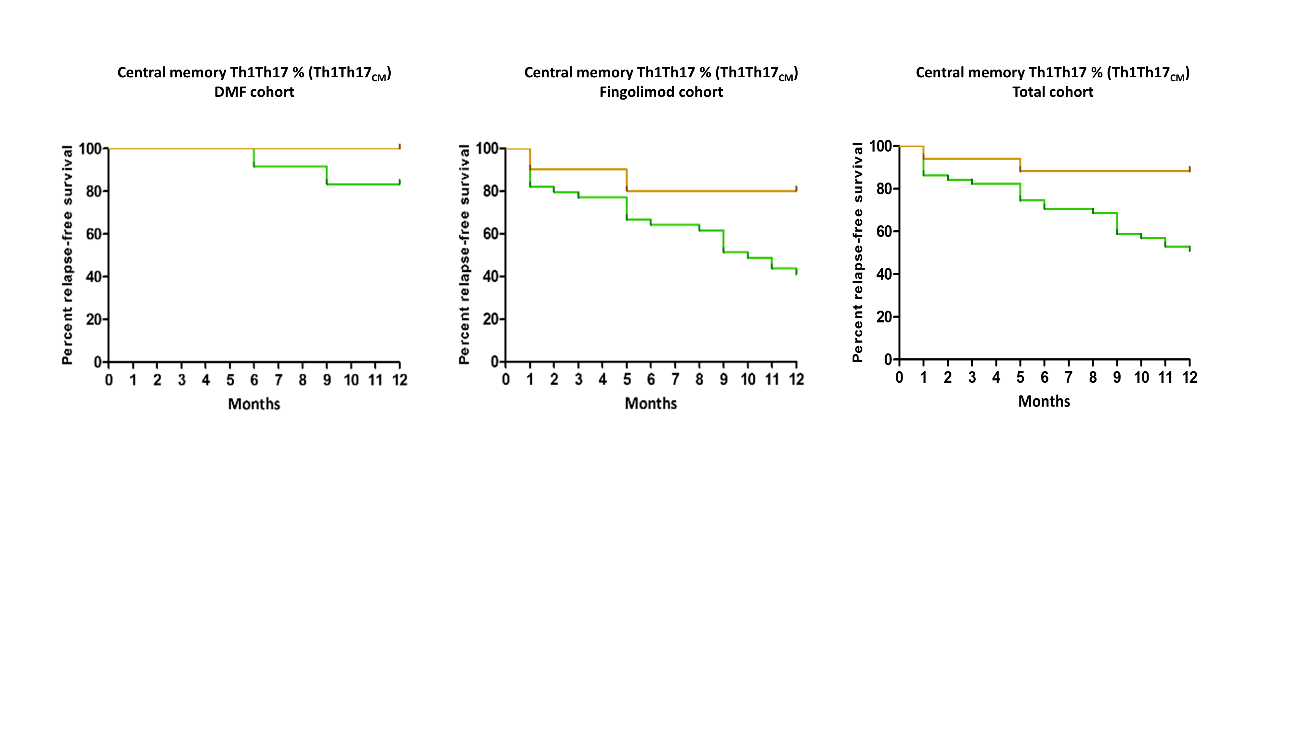


a)

b)

c)

**Figure S2. Relapse-free survival curve for patients during the first 12 months DMF treatment (a), fingolimod treatment (b) and total cohort of patients (c).** Groups were separated by percentage of Th1Th17_CM_ out of CD4^+^ T cells. Patients with less than 11.48% of Th1Th17_CM_ (n= 26) and more than 11.48 % (n=27) ((a) p=0.27; (b) p=0.08; (c) p<0.01, log-rank test).
